# Disease-specific differences in gene expression, mitochondrial function and mitochondria-endoplasmic reticulum interactions in iPSC-derived cerebral organoids and cortical neurons in schizophrenia and bipolar disorder

**DOI:** 10.1007/s44192-023-00031-8

**Published:** 2023-03-09

**Authors:** Annie Kathuria, Kara Lopez-Lengowski, Donna McPhie, Bruce M. Cohen, Rakesh Karmacharya

**Affiliations:** 1 Center for Genomic Medicine, Massachusetts General Hospital, 185 Cambridge Street, Boston, MA 02114 USA; 2 Chemical Biology Program, Broad Institute of MIT & Harvard, Cambridge, MA USA; 3 Department of Psychiatry, Harvard Medical School, Boston, MA USA; 4 Schizophrenia and Bipolar Disorder Program, McLean Hospital, Belmont, MA USA; 5 Program in Neuroscience, Harvard University, Cambridge, MA USA; 6 Program in Chemical Biology, Harvard University, Cambridge, MA USA; 7 Harvard Stem Cell Institute, Cambridge, MA USA

## Abstract

We compared transcriptomic profiles of cerebral organoids differentiated from induced pluripotent stem cells of eight schizophrenia and eight bipolar disorder patients to identify genes that were differentially expressed in cerebral organoids between two disorders. Gene ontology analysis showed relative up-regulation in schizophrenia organoids of genes related to response to cytokines, antigen binding and clathrin-coated vesicles, while showing up-regulation in bipolar disorder of genes involved in calcium binding. Gene set enrichment analysis revealed enrichment in schizophrenia of genes involved in mitochondrial and oxidative phosphorylation while showing enrichment in bipolar disorder of genes involved in long term potentiation and neuro-transporters. We compared mitochondrial function in cerebral organoids from schizophrenia and bipolar disorder subjects and found that while schizophrenia organoids showed deficits in basal oxygen consumption rate and ATP production when compared to healthy control organoids, while bipolar disorder organoids did not show these deficits. Gene ontology analyses also revealed enrichment in bipolar disorder of genes in ion binding and regulation of transport. Experiments examining the interaction between mitochondria and endoplasmic reticulum in cortical neurons from bipolar disorder subjects showed a significantly lower number of contact sites between mitochondria and endoplasmic reticulum when compared to cortical neurons from schizophrenia patients. These results point to disease-specific deficits in mitochondrial respiration in schizophrenia and in mitochondrial-endoplasmic reticulum interactions in bipolar disorder.

**Supplementary Information:**

The online version contains supplementary material available at 10.1007/s44192-023-00031-8.

## Introduction

Schizophrenia (SCZ) and bipolar I disorder (BPI) are amongst the most severe psychiatric disorders, with significant disease burden in the global context. In its prototypical presentation, schizophrenia is characterized as a thought disorder, with disturbances in the realms of perception and cognition, including positive symptoms (i.e. hallucinations, delusions), negative symptoms (i.e. apathy, avolition, affective flattening) and cognitive deficits (i.e. working memory, executive functioning) and is a chronic condition. Bipolar disorder, on the other hand, is primarily a mood disorder, characterized by the two poles of mania and depression and usually recurrent. Clinical, neuropathological, neuroimaging and genetic studies show that there are shared facets as well as distinct differences between the two disorders [1, 2]. Genome-wide association studies (GWAS) of subjects with schizophrenia and bipolar disorder have revealed shared genetic susceptibilities though there are distinct risk variants for each of the disorders as well [3, 4]. With regards to treatment, antipsychotic medications are the mainstay for the treatment of schizophrenia but a number of antipsychotic medications also have high efficacy in the treatment for manic and depressive phases of bipolar disorder [5]. However, the converse does not hold true—mood stabilizers do not have high efficacy in the treatment of schizophrenia, especially in monotherapy [6]. Research aimed at delineating the shared and distinct biological features of these two disorders can provide insights into the neurobiological underpinnings of these disorders.

Schizophrenia and bipolar disorder have historically been treated as disorders with distinct symptoms and clinical courses, starting with the designation of dementia praecox and manic-depressive insanity by Emil Kraepelin over a century ago [2]. Dementia praecox was later coined as schizophrenia by Eugen Bleuler while manic-depressive insanity is now more commonly referred to as bipolar disorder. However, there is convergent evidence from genetic, clinical and neuropathological studies that indicate some shared biology and some unique facets between the two disorders [1, 2]. Studies examining gene expression in post-mortem brains had also revealed both shared features as well as distinct differences between schizophrenia and bipolar disorder [7]. Transcriptome analysis of post-mortem tissue from the anterior cingulate cortex of schizophrenia and bipolar disorder subjects showed some correlation in gene expression differences in schizophrenia and bipolar disorder that indicated shared biology related to dysregulation of cytoskeleton remodelling and lysosomal function as well as shared genes involved in synaptic biology, adherens junctions, mitochondrial biology and biosynthetic metabolic processes [8]. However, gene expression data from cortical brain tissues showed that while the transcriptome profiles schizophrenia and autism are significantly correlated, there was no statistically significant correlation between the schizophrenia and bipolar disorder transcriptome profiles [9].

Induced pluripotent stem cells (iPSCs) that can be reprogrammed from readily accessible tissue from human subjects have enabled new ways to study human brain development and provided methods to generate and study human neuronal cells in the laboratory [10–13]. Human iPSCs can be differentiated to generate a range of neuronal and glial cells relevant to disease biology of schizophrenia and bipolar disorder [14–17]. In addition to two-dimensional neuronal cultures, advances in tissue engineering now enable generation of human three-dimensional cerebral organoids from the iPSCs [18]. These cerebral organoids recapitulate human cortical development and contain many neuronal and glial subtypes, including cortical neuron subtypes, synapses and exhibiting electrophysiological activity [19].

We had previously undertaken RNA-seq experiments to compare gene expression profiles between cerebral organoids from iPSCs of schizophrenia subjects and healthy subjects as well as between cerebral organoids from iPSCs of bipolar disorder subjects and healthy subjects [20, 21]. Analyses of the transcriptomic data showed that both schizophrenia and bipolar disorder cerebral organoids have gene expression differences in pathways involved in neurodevelopment and in tissue generation, when compared to the gene expression profiles of cerebral organoids from healthy subjects [20, 21]. Here, we used the raw RNA-seq data from these experiments to undertake a head-to-head comparison of the transcriptome profiles of cerebral organoids from eight schizophrenia subjects and eight bipolar disorder subjects, with an eye towards identifying cellular pathways that were differentially regulated between these two disorders. In the head-to-head comparison, we found that cerebral organoids from schizophrenia subjects showed relative up-regulation, compared to bipolar disorder organoids, of genes involved in response to immune system and in mitochondrial function while cerebral organoids from bipolar disorder subjects showed relative up-regulation of genes involved in calcium signaling and in neuro-transporters. As a follow-up to the transcriptomic data, we had previously undertaken mitochondrial function studies in schizophrenia organoids and examined mitochondria-endoplasmic reticulum (ER) interactions in iPSC-derived cortical neurons in bipolar disorder [20, 21]. Here, report on additional experiments investigating mitochondrial function in bipolar disorder organoids and new data generated to analyze mitochondria-ER interactions in schizophrenia iPSC-derived cortical neurons. We compared our new data with the previously published findings so that we could delineate which cellular features are distinct in the two disorders. We found that schizophrenia organoids, but not bipolar disorder organoids, exhibit dysfunction in basal respiration rate and ATP production. Conversely, experiments measuring mitochondria-endoplasmic reticulum (ER) interactions in iPSC-derived cortical neurons showed a relative decrease in ER-mitochondria contact sites in bipolar disorder neurons when compared to schizophrenia and healthy control neurons.

## Materials and methods

### Differentiation of excitatory cortical neurons

The iPSC cultures were maintained in NutriStem hPSC XF Medium (Biological Industries, 01-0005). When iPSCs reached confluency, the cells were differentiated into excitatory neurons on Geltrex (ThermoFisher, A1413202) coated plates. Cells were maintained in N2/B27 media containing 485 mL Neurobasal medium (Life Technologies), 5 mL N2 (Gibco, 17502001), 5 mL Glutamax (ThermoFisher Scientific), 5 mL penicillin–streptomycin), 10 mL B27 supplement (Gibco, 17504044). For the first 7 days, the medium was supplemented with 10 μM SB431542 (Sigma-Aldrich, S4317), 1 μM dorsomorphin (Sigma-Aldrich, P5499) and 100 nM LDN193189 (Sigma-Aldrich, SML0559). On days 8–29, the N2/B27 media was changed daily, without supplementing the media with SB431542, dorsomorphin or LDN193189. On day 30, cells were transitioned in Brainphys Neuronal Medium (Stemcell Technologies, 05790) supplemented with B-27, with media change twice weekly. Methods for differentiation of cerebral organoids and extraction of RNA had been described in our previous studies [19, 20].

### RNA-seq experiments and data processing

We used the Illumina RiboZero TruSeq Stranded Total RNA Library Prep Kit (Illumina) to construct the RNA-seq library and used the Illumina NovaSeq6000 platform for sequencing in the 100 nt, paired-end configuration, as described previously [20, 21]. We obtained an average of 60 million reads for each sample. The reads were trimmed with Cutadapt and aligned to the reference genome (hg38 UCSC assembly) to analyze gene expression using TopHat v2.0.14 and Bowtie v2.10 with default parameters and RefSeq annotation (genome-build GRCh38.p9) [22]. We used Cufflinks v2.2.1 to analyze distribution of alignments and quantile normalized FPKM (fragments per kilobase of exon model per million reads mapped) values [23, 24]. We utilized Cuffdiff v2.2.1 to perform differential expression testing. We did not consider sex differences for this analysis. The false discovery rate (FDR) was 0.05. The raw data analysis is included in Supplementary file 1, in sheet 3 titled significant genes. Tables 1, 2 and 3 show q values represent FDR-adjusted p-value of the test statistic. RT-PCR was used to validate a number of key relevant genes.

**Table 1:** Gene set enrichment analysis

	Size	ES	NES	NOM p-val	FDR q-val	FWER p-val	Rank at max	Leading edge
Reactome								
Reactome_Influenza_Infection	154	− 0.59132	− 2.80241	0	0	0	10,503	tags = 73%, list = 27%, signal = 99%
Reactome_Mitochondrial_Translation	93	− 0.60753	− 2.62088	0	0	0	13,391	tags = 86%, list = 34%, signal = 131%
Reactome_Respiratory_Electron_Transport	89	− 0.60034	− 2.61611	0	0	0	12,856	tags = 84%, list = 33%, signal = 125%
Reactome_Infectious_Disease	371	− 0.49008	− 2.60519	0	0	0	13,579	tags = 68%, list = 35%, signal = 103%
Reactome_Cell_Cycle_Checkpoints	282	− 0.51048	− 2.60441	0	0	0	15,024	tags = 74%, list = 39%, signal = 120%
Reactome_Naplus_Cl_Dependent_Neurotransmitter_Transporters	19	0.515474	1.541765	0.038388	0.66119	1	1466	tags = 32%, list = 4%, signal = 33%
Reactome_Role_Of_Phospholipids_In_Phagocytosis	33	0.452627	1.529319	0.032136	0.623584	1	4477	tags = 24%, list = 11%, signal = 27%
Reactome_Plasma_Lipoprotein_Assembly	19	0.504823	1.504998	0.043222	0.652722	1	2605	tags = 32%, list = 7%, signal = 34%
Reactome_Xenobiotics	24	0.466743	1.485898	0.049904	0.666401	1	8655	tags = 50%, list = 22%, signal = 64%
Reactome_Long_Term_Potentiation	23	0.474369	1.470371	0.046693	0.608513	1	8104	tags = 48%, list = 21%, signal = 60%
Hallmark								
Hallmark_Myogenesis	199	− 0.55399	− 2.73616	0	0	0	5986	tags = 43%, list = 15%, signal = 51%
Hallmark_E2F_Targets	198	− 0.51894	− 2.56449	0	0	0	13,107	tags = 65%, list = 34%, signal = 98%
Hallmark_Oxidative_Phosphorylation	185	− 0.52698	− 2.53241	0	0	0	13,844	tags = 71%, list = 36%, signal = 109%
Hallmark_Unfolded_Protein_Response	111	− 0.43549	− 1.96044	0	0	0	14,339	tags = 65%, list = 37%, signal = 102%
Hallmark_Glycolysis	198	− 0.38222	− 1.88766	0	0	0	13,694	tags = 57%, list = 35%, signal = 87%
Hallmark_Pancreas_Beta_Cells	40	0.268541	0.960266	0.515539	1	1	2039	tags = 10%, list = 5%, signal = 11%
Hallmark_Spermatogenesis	132	0.17472	0.771267	0.937394	1	1	9809	tags = 27%, list = 25%, signal = 36%
Hallmark_Xenobiotic_Metabolism	200	0.164059	0.76698	0.98087	0.93739	1	5051	tags = 12%, list = 13%, signal = 13%
KEGG								
KEGG_Ribosome	86	− 0.66314	− 2.80034	0	0	0	10,890	tags = 93%, list = 28%, signal = 129%
KEGG_Parkinsons_Disease	99	− 0.50716	− 2.26377	0	0	0	13,694	tags = 75%, list = 35%, signal = 115%
KEGG_Oxidative_Phosphorylation	100	− 0.51326	− 2.26148	0	0	0	13,763	tags = 71%, list = 35%, signal = 109%
KEGG_Small_Cell_Lung_Cancer	84	− 0.50382	− 2.18709	0	0	0	8917	tags = 50%, list = 23%, signal = 65%
KEGG_P53_Signaling_Pathway	67	− 0.52866	− 2.14187	0	9.17E−04	0.003	12,063	tags = 64%, list = 31%, signal = 93%
KEGG_Taste_Transduction	51	0.457469	1.688169	0.001692	0.234029	0.361	7615	tags = 49%, list = 20%, signal = 61%
KEGG_Linoleic_Acid_Metabolism	28	0.510943	1.677292	0.011858	0.129439	0.388	8626	tags = 43%, list = 22%, signal = 55%
KEGG_Type_I_Diabetes_Mellitus	42	0.433637	1.558308	0.012797	0.188571	0.766	8960	tags = 43%, list = 23%, signal = 56%
KEGG_Asthma	29	0.486376	1.629667	0.013619	0.132661	0.538	8960	tags = 52%, list = 23%, signal = 67%
KEGG_Retinol_Metabolism	64	0.358277	1.385493	0.043956	0.410179	0.994	7681	tags = 28%, list = 20%, signal = 35%

Gene set enrichment analysis (GSEA) data for DEGs using three different databases (Hallmark, KEGG, Reactome) tabulated according to normalized enrichment score (NES) and False discovery rate (FDR) q-value. Positive correlation indicates relative association with gene expression in bipolar disorder and a negative correlation indicates relative association with gene expression in schizophrenia

**Table 2:** Mitochondrial genes upregulated in schizophrenia compared to bipolar disorder

Gene	Log2(fold_change)	P_value	Q_value
B2M	inf	0.00005	0.0168915
SFTPB	5.47445	0.00005	0.0168915
P2RX3	4.09903	0.00015	0.041525
PKHD1L1	4.00139	0.00005	0.0168915
THSD7B	3.9018	0.00005	0.0168915
EBF2	3.73444	0.00005	0.0168915
ACTA1	3.71003	0.00005	0.0168915
SHOX2	3.54632	0.00005	0.0168915
BCL6B	3.40011	0.00005	0.0168915
MUC5B	3.27599	0.00005	0.0168915
CD5	3.27526	0.00005	0.0168915
CASQ2	3.27468	0.00005	0.0168915
MYBPC2	3.20693	0.00005	0.0168915
NPTX2	3.17192	0.0001	0.0297493
NHLH1	3.15736	0.00005	0.0168915
MYBPH	3.05216	0.00005	0.0168915
ASB4	3.01621	0.0001	0.0297493
MUC12	2.87732	0.00005	0.0168915
SHD	2.83455	0.00005	0.0168915
TRIM55	2.73832	0.00005	0.0168915
ACTC1	2.72941	0.00005	0.0168915
MYH3	2.66443	0.00005	0.0168915
EBF1	2.6039	0.00005	0.0168915
CHRND	2.60343	0.00005	0.0168915
APLN	2.54672	0.00005	0.0168915
GRID2	2.50442	0.0002	0.0480289
MYL4	2.42199	0.0001	0.0297493
KRT1	2.38926	0.0002	0.0480289
TNNT2	2.26791	0.00005	0.0168915
NEFM	2.25293	0.00005	0.0168915
AFAP1L1	2.2312	0.00005	0.0168915
EYA1	2.10989	0.0002	0.0480289
RBM24	2.05014	0.0002	0.0480289
RYR1	2.00653	0.00005	0.0168915
C7	1.92159	0.00005	0.0168915
COL19A1	1.87283	0.0001	0.0297493
DUOX2	1.86384	0.0001	0.0297493
SERPINA3	1.79983	0.00005	0.0168915
TNNC1	1.798	0.0002	0.0480289
NTRK2	1.73311	0.00005	0.0168915
CAPN6	1.60497	0.00005	0.0168915
NEUROD1	1.53609	0.00005	0.0168915
MCAM	1.52598	0.00005	0.0168915
SPOCK2	1.43396	0.00005	0.0168915
ARHGAP29	1.29965	0.00005	0.0168915
CDR1	1.0788	0.0001	0.0297493
PEG10	1.07848	0.0002	0.0480289
FREM2	1.00581	0.0002	0.0480289

List of mitochondria-associated genes in the MitoCarta 2.0 database that were upregulated in SCZ organoids vis-à-vis BPI organoids and in BPI organoids vis-à-vis SCZ organoids

**Table 3:** Mitochondrial genes upregulated in bipolar disorder compared to schizophrenia

Gene	Log2(fold_change)	P_value	Q_value
CCL25	4.42404	0.00005	0.0168915
GIP	4.04306	5.00E−05	0.0168915
HLA-DRB1	3.66592	0.00005	0.0168915
OPRK1	3.58192	0.00005	0.0168915
CX3CR1	3.33744	0.00005	0.0168915
ASAH2	3.32305	0.00005	0.0168915
LCT	3.24615	0.00005	0.0168915
APOC3	3.05208	0.00005	0.0168915
SLC2A2	2.78622	0.0001	0.0297493
FOLH1	2.75393	0.00005	0.0168915
GATA4	2.37677	0.0002	0.0480289
ANXA13	2.13002	0.00005	0.0168915
COL2A1	1.98566	0.00005	0.0168915
SI	1.92321	0.00005	0.0168915
PIGR	1.76004	0.00005	0.0168915
SLC5A1	1.70496	0.0001	0.0297493
APOB	1.69844	0.00005	0.0168915
SULT2A1	1.69654	0.0002	0.0480289
MTTP	1.66128	0.00005	0.0168915
MALRD1	1.6595	0.00005	0.0168915
OLFM4	1.61724	0.00005	0.0168915
GSTA1	1.3839	0.00005	0.0168915
OAT	1.24558	0.00015	0.041525
FOS	1.2401	0.00005	0.0168915
PRLR	1.21405	0.00015	0.041525
XDH	1.21051	0.00005	0.0168915
MME	1.07592	0.00005	0.0168915

### Gene ontology and gene set enrichment analyses

We used Kyoto Encyclopedia of Genes and Genomes (KEGG) analysis on all differentially regulated genes, utilizing the Functional Enrichment Analysis unit of HOMER v.3. [25]. The reference list for the GO analysis is from Clarivate-MetaCore + MetaDrug™ version 19.1 build 69600. In the figures, we have listed the genes that reached significance (p < 0.05). We performed gene set enrichment analysis (GSEA) with default parameters using the GSEA software for all expressed genes with FPKM values calculated by Cufflinks against following data sets v6.2: Hallmark, REACTOME and KEGG [26]. We used Human MitoCarta 2.0 (https://www.broadinstitute.org/files/shared/metabolism/mitocarta/human.mitocarta2.0.html), was used to identify DEGs implicated in human mitochondrial pathways, as shown in Tables 2 and 3.

### Mitochondrial respiration—seahorse mito stress test assay

We used the Seahorse XF Cell Mito Stress Test (Agilent, 103015–100) to assess mitochondrial respiration in cerebral organoids that had been grown for 9 months, as described previously for the comparison between healthy control and schizophrenia organoids [20]. We undertook the Seahorse assay for the bipolar disorder organoids as well using the same assay in order to compare the schizophrenia and bipolar disorder organoids. Cerebral organoids were cut into three sections and plated on Seahorse XFe96 Spheroid microplates (Agilent, 102978–100) coated with 10 μg/mL laminin (Sigma-Aldrich, L2020). Effects of the following perturbations were recorded sequentially: 2 mM oligomycin, 2 mM FCCP (Carbonyl cyanide 4-(trifluoromethoxy)phenylhydrazone), and 2 mM rotenone and 2 mM antimycin + 2 mM rotenone. Data were analyzed with Wave software 2.4 (Agilent). All organoids used in this assay were generated at the same time.

### Proximity ligation assay

The proximity ligation assay (PLA) was used to quantify contact points between the endoplasmic reticulum (ER) and mitochondria in the human iPSC-derived cortical neurons, using the Duolink In Situ PLA Probe (Sigma-Aldrich DUO92002, DUO92004) [27]. We had described the experimental protocol previously in comparing PLA in cortical neurons from bipolar disorder iPSCs and healthy control iPSCs [21]. We collected additional data on PLA in cortical neurons from schizophrenia iPSCs as well to compare with the previously reported PLA data in cortical neurons differentiated from bipolar disorder and healthy control cortical iPSCs. Cortical neurons that had been differentiated for 90 days were fixed with 4% PFA for 20 min, washed in PBS three times and then permeabilized in 0.1% Triton-X100 for 15 min. The samples were then incubated in the blocking solution for 30 min at 37 °C. The following primary antibodies, diluted in PBS, were added to the cells and incubated overnight at 4 °C: anti-VDAC1 antibody [20B12AF2] (Abcam, ab14734, diluted 1:100) and anti-IP3R1 antibody (Abcam, ab5804, diluted 1:500) [27]. The antibodies were then removed and the cells washed with PBS + 0.01% Triton-X100. The PLA probes, which were diluted 1:5 in the provided diluent, were added to the cells and incubated at 37 °C for 1 h. Ligation and amplification were undertaken using the Duolink® In Situ Detection Reagent, with ligation stock diluted 1:5 in high-purity water and the ligase diluted 1:40 and vortexed before being added to the samples. After 30 min incubation at 37 °C, the samples were washed twice with TBS-T. The amplification stock solution was diluted 1:5 in high-purity water and polymerase was diluted 1:80 in the amplification solution and vortexed before being added to the samples and incubated for 100 min at 37 °C. The samples were washed in 1 × saline sodium citrate (SSC) washing buffer for 2 min, followed by a wash in 0.01 × SSC washing buffer for another 2 min. Prolong Gold Antifade Mount with DAPI (Life Technologies, P36931) was then added and neurons which were imaged at 60× resolution with the PerkinElmer Opera Phoenix High-Content Screening System. The Opera Phoenix and Harmony software (Perkin Elmer) were used to quantify the number of PLA positive spots.

## Results

### Gene expression analysis of cerebral organoids from schizophrenia and bipolar disorder subjects

We used bulk RNA-seq data from iPSC-derived cerebral organoids from iPSCs of eight patients each with bipolar disorder and schizophrenia, as described in earlier studies [20, 21]. These cerebral organoids developed along the telencephalic lineage to continue to ~ 5 mm after 7 months. Cerebral organoids generated from iPSC lines different groups showed no gross differences between them and all groups showed a similar proportion of different cell types and expression of a range of neuronal and glial markers, including MAP2, Ctip2, Satb2, Pax6, TBR2, Cux1, LHX6, glutamine synthetase (GS), GFAP, oligodendrocytes-specific protein/claudin11 (OSP), myelin basic protein (MBP) and IBA1 [20, 21]. Overall, these results show overall consistency in the cellular composition of organoids generated from different lines.

**Fig. 1:**
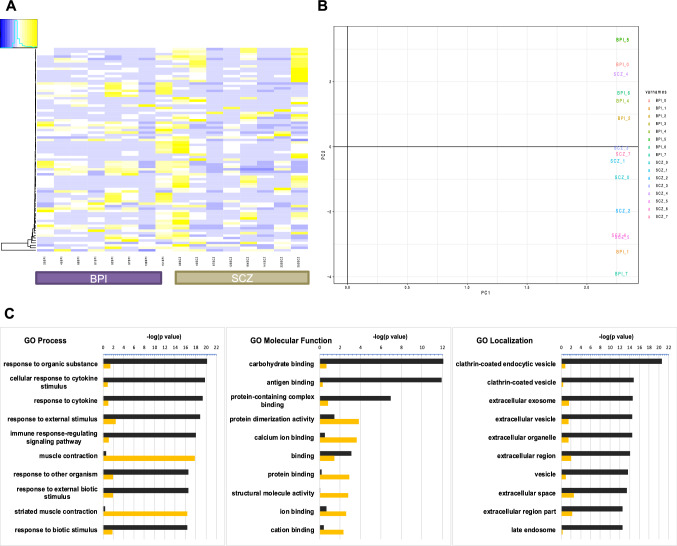
Comparative gene expression analysis of cerebral organoids differentiated from iPSCs of subjects with schizophrenia and bipolar disorder. **A** Heatmaps of differentially expressed genes (DEGs) of 6-month-old cerebral organoids generated from iPSCs of eight SCZ and eight BPI subjects. **B** Principal Component Analysis (PCA) of DEGs of SCZ and BPI cerebral organoids. **C** Gene ontology (GO) analysis of DEGs for SCZ vs BPI. GO analyses for biological process, molecular function and cellular component/protein localization are rank-ordered according to significance (*p* value) for the top 10 categories—categories up-regulated in SCZ are depicted in black and categories up-regulated in BPI are depicted in orange

The heatmap depicting the differentially expressed genes (DEGs) in the RNA-seq experiments showed similar gene expression patterns between the two disease groups. (Fig. 1A). With principal component analysis, we assessed line-to-line and group-to-group variability and found that the gene expression data revealed overlap between schizophrenia and bipolar disorder (Fig. 1B). There were 83 significant DEGs between schizophrenia and bipolar disorder, out of which 53 genes were up-regulated in schizophrenia compared to bipolar disorder and 30 genes that were up-regulated in bipolar disorder compared to schizophrenia. Gene ontology analysis on these genes (Fig. 1C) revealed that schizophrenia organoids show relative up-regulation of genes involved in antigen binding, cytokine response and response to organic substances while the bipolar disorder organoids show relative up-regulation of genes involved in calcium binding, ion binding and muscle contraction. These analyses show that pathways representing genes that are up-regulated in schizophrenia organoids are enriched immune signaling processes while pathways representing genes that are up-regulated in bipolar disorder organoids are enriched for ion channel biology.

### Gene ontology

**Fig. 2:**
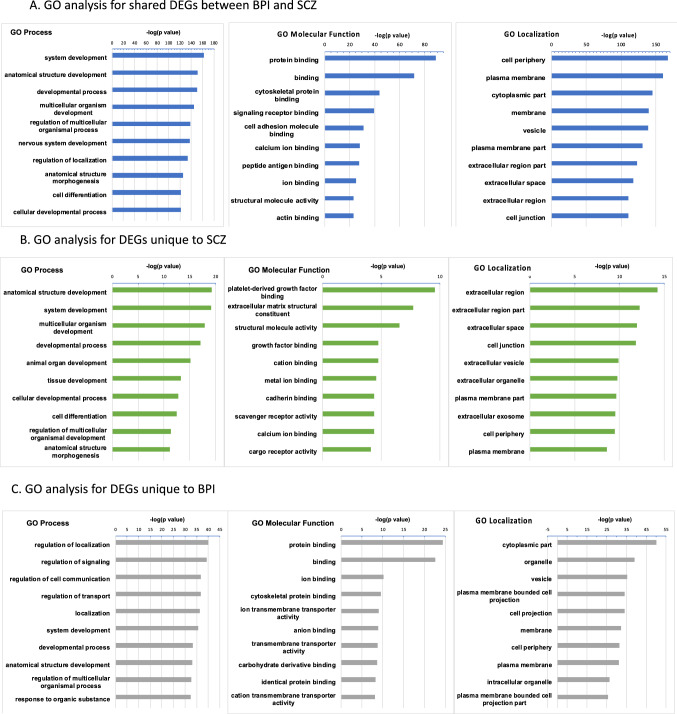
Gene ontology analysis. **A** GO analysis of biological process, molecular function and cellular component/protein localization for DEGs that were shared between SCZ and BPI cerebral organoids when compared to CON cerebral organoids, rank-ordered according to significance (*p* value) for the top 10 categories. **B** GO analysis of biological process, molecular function and cellular component/protein localization for DEGs that were unique to SCZ cerebral organoids, rank-ordered according to significance (*p* value) for the top 10 categories. **C** GO analysis of biological process, molecular function and cellular component/protein localization for DEGs that were unique to BPI, rank-ordered according to significance (*p* value) for the top 10 categories

We categorized DEGs into the following three categories: (i) genes that were differentially regulated in the same manner in both schizophrenia and bipolar disorder when compared to healthy control organoids, (ii) genes that were differentially regulated in schizophrenia compared to healthy control but not in bipolar disorder and (iii) genes that were differentially regulated in bipolar disorder relative to healthy control but not in schizophrenia. We sought to delineate biological pathways that were similarly affected in the two disorders as well as pathways that were specific for the particular disease. We performed gene ontology (GO) analyses after rank-ordering the top hits according to significance (*p-*value) (Fig. 2A–C). Among significant GO: Biological Process categories that were differentially regulated in both schizophrenia and bipolar disorder were pathways involved in development, including nervous system development (Fig. 2A). GO: Localization analysis showed shared differences in cellular component/protein localization categories of cell periphery, plasma membrane cytoplasmic part and vesicles (Fig. 2A). The GO: Molecular Function analysis showed shared differences in schizophrenia and bipolar disorder in categories involving cytoskeletal protein binding, cell adhesion and peptide antigen binding (Fig. 2A).

When gene ontology analyses were undertaken with DEGs unique to schizophrenia, the most significant GO: Biological Process categories included developmental processes while the significant GO: Localization categories showed differences in extracellular regions (Fig. 2B). The significant GO: Molecular Function categories indicated differences in schizophrenia in biology involving platelet-derived growth factor binding, extracellular matrix and cadherin binding (Fig. 2B). Similar analysis of DEGs unique to bipolar disorder for the significant GO: Biological Process categories showed differences in regulation of localization and signaling while the significant GO: Localization analysis shows significant differences in categories related to cytoplasm, organelles and vesicles (Fig. 2C). The significant GO: Molecular Function categories in bipolar disorder included a number of protein and ion binding as well as categories involving transporter activity (Fig. 2C).

### Gene set enrichment analysis

We carried out gene set enrichment analysis (GSEA) using three datasets (Hallmark, KEGG, Reactome) to identify gene-enriched pathways in DEGs for one disorder vis-à-vis the other. GSEA is an analytical method that allows us to examine gene expression data and determine whether a collated set of genes, annotated based on their common roles in various biological processes, show significant differences between two groups [26]. We tabulated positive and negative correlation with schizophrenia and bipolar disorder according to the normalized enrichment scores (Table 1). Analysis of DEGS with relative enrichment in schizophrenia cerebral organoids showed involvement of pathways involved in inflammatory response (Reactome), oxidative phosphorylation (KEGG, Hallmark) and mitochondrial function (Reactome) in all three pathway databases (Table 1). Analysis of pathways for DEGs with relative enrichment in bipolar disorder cerebral organoids showed involvement of categories including neuro-transporters (Reactome) and long-term potentiation (Reactome) (Table 1). These results point to disease-specific differences related to a number of pathways, including in mitochondrial function in schizophrenia.

### Functional analysis of schizophrenia and bipolar disorder organoids

**Fig. 3:**
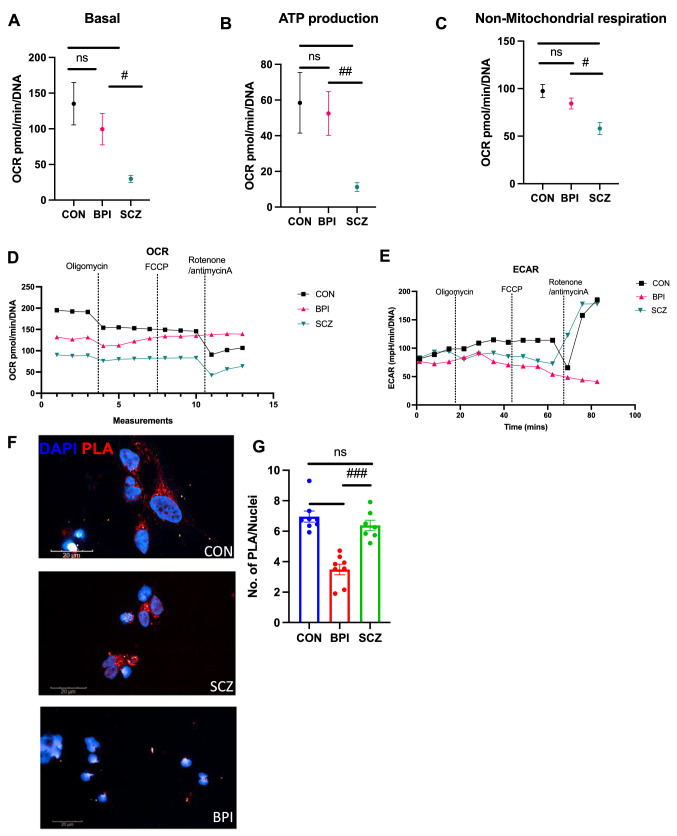
Functional analysis of mitochondrial respiration in iPSC-derived cerebral organoids and mitochondrial-ER interactions in iPSC-derived cortical neurons. **A**–**C** Seahorse Cell Mito Stress Test assay conducted with 9-month-old cerebral organoids. Data collected from cerebral organoids differentiated from eight BPI iPSC lines and compared with data from SCZ and CON organoids reported previously [20]. Oxygen consumption rate (OCR) results for basal rate, ATP production, and non-mitochondrial oxygen consumption rate are shown, along with values for extracellular acidification rate (ECAR). Data collected from three sections in each condition and values shown as mean ± SEM. Normality was tested via Kolmogorov–Smirnov, but since all data were not normally distributed, Mann–Whitney test was performed. Basal: CON vs SCZ ***p = 0.0001, SCZ vs BPI ^#^p = 0.0249. ATP production: CON vs SCZ **p = 0.0012, SCZ vs BPI ^##^p = 0.0016. Non-mitochondrial oxygen consumption rate: CON vs SCZ **p = 0.0003, SCZ vs BPI ^#^p = 0.0237. CON vs BPI was insignificant in all categories. **D** Mitochondrial OCR (oxygen consumption rate) graph. Šídák's multiple comparisons test: CON vs SCZ ***p = 0.0001, CON vs BPI p = ns, SCZ vs BPI ***p = 0.0001. **E** ECAR (extracellular acid reflux rate) graph. Šídák's multiple comparisons test, performed CON vs SCZ, p = ns, CON vs BPI ***p = , CON vs BPI ***p = 0.0010 and SCZ vs BPI *p = 0.0102. **F** Representative images of proximity ligation assay (PLA) in CON, SCZ and BPI cortical neurons. Scale bar: 20 μM. **F** Quantification of PLA positive objects/nuclei. Data shown from cortical neurons generated from eight SCZ iPSC lines and compared with PLA data from eight CON and eight BPI iPSC lines reported previously [21]. Values are shown as mean ± SEM. Two-tailed Mann–Whitney U test: CON vs BPI ***p = 0.0002, SCZ vs BPI ^###^p = 0.0003, CON vs SCZ was insignificant

The GSEA analysis revealed significant differences in schizophrenia and bipolar disorder in categories of mitochondrial translation, respiratory electron transport and oxidative phosphorylation. Furthermore, we compared the DEGs from the bipolar disorder vs schizophrenia analysis to Human MitoCarta 2.0 database and found that 75 genes among the 83 DEGS were implicated in human mitochondrial pathways—genes that had relative up-regulation in schizophrenia are shown in Table 2 while genes that had relative up-regulation in bipolar disorder are shown in Table 3. To follow up on these observations, we undertook mitochondrial function experiments with the Seahorse Cell Mito Stress Test in cerebral organoids from bipolar disorder lines and compared them with the Seahorse Cell Mito Stress Test in cerebral organoids from schizophrenia lines that we had reported on previously [20]. These experiments involved live-cell metabolic experiments with 9-month-old cerebral organoids, in the setting of exposure to specific annotated perturbations in the assay. While the schizophrenia organoids had significantly lower basal oxygen consumption rate, ATP production and non-mitochondrial oxygen consumption when compared to healthy control organoids as we had reported earlier [20], the bipolar disorder organoids showed no significant differences in this regard (Fig. 3A–C). There was significant difference in the oxygen consumption rate (OCR) between schizophrenia and healthy control organoids in the setting of different perturbations, but these differences were also not observed in bipolar disorder organoids (Fig. 3D). However, bipolar disorder organoids showed lower ECAR rate which suggests lower rate of glycolysis when compared to healthy control and schizophrenia organoids (Fig. 3E).

Mitochondria–endoplasmic reticulum interactions have been hypothesized to be abnormal in mood disorders [28, 29]. The contact sites between endoplasmic reticulum (ER) and mitochondria, called mitochondria-associated membranes (MAMs), facilitate Ca^2+^ exchange between the two organelles and play important roles in mitochondrial fission and fusion, intracellular trafficking, calcium homeostasis, ER stress and unfolded proteins, phospholipid exchange as well as autophagy and inflammation [30]. Since the GO analysis had indicated enrichment in bipolar disorder of genes involved in ion binding and regulation of transport (Figs. 1C and 2C), we sought to investigate if there were disease-specific differences in MAMs between schizophrenia and bipolar disorder. In a previous study, we had shown that bipolar disorder cortical neurons showed a significant reduction in MAMs when compared to healthy control cortical neurons, with data obtained using the proximity ligation assay to quantify the number of contact sites between the mitochondria and ER [21]. We collected additional data on MAMs in cortical neurons from schizophrenia iPSCs and compared them with the previous data from bipolar neurons. We found that the control and schizophrenia neurons were similar in terms of MAMs in the cortical neurons but only the bipolar disorder cortical neurons showed a significant reduction in MAMs (Fig. 3F and G).

## Discussion

Patient-derived cells provide live biological material that can be utilized to interrogate disease biology relevant to schizophrenia and bipolar disorder [13, 31, 32]. Recently iPSC-derived neuronal and glial cells have been utilized to examine gene expression differences in schizophrenia and bipolar disorder [33, 34]. While most of the early gene expression studies had focused on the use of neural progenitor cells and two-dimensional neuronal cultures, three-dimensional cerebral organoids are also being utilized for the study of disease biology, including examination of transcriptomic profiles [35, 36]. In our study comparing the similarities and differences in transcriptomic profiles of schizophrenia and bipolar disorder organoids, we found shared categories related to nervous system development in these two disorders. While there is significant evidence lending support to neurodevelopmental abnormalities in schizophrenia, the evidence for neurodevelopmental processes in bipolar disorder is not as well established [37]. In the ex vivo models that we studied which recapitulate neurodevelopmental processes, the findings suggest a shared feature related to deviation from normal development as captured by the gene expression patterns.

While we had previously examined transcriptomic differences in schizophrenia and bipolar disorder when compared to healthy control organoids, we undertook this additional study to examine what genes and pathways are differentially regulated in schizophrenia vs. bipolar disorder. While some of the biological and clinical features are shared between schizophrenia and bipolar disorder, it is hypothesized there are distinct differences in the neurobiology of the two disorders as well. By undertaking a head-to-head comparison, we hoped to delineate genes and pathways that distinguish the underlying biology in these two disorders. When examining the gene ontology patterns of genes that were differentially regulated in schizophrenia organoids *vis-a-vis* bipolar disorder organoids, we found that the differentially expressed genes in schizophrenia were enriched for pathways involved in immune signaling. This is intriguing given the convergent evidence for the role of the immune system dysregulation in schizophrenia [38]. Brain organoids generated from iPSCs generate a range of neuronal and glial cell types, including microglia, and some of the differences in schizophrenia organoids may result from differences in neuro-immune processes in the context of the developing brain [21]. Another category that showed distinct enrichment in schizophrenia cerebral organoids vis-à-vis bipolar disorder organoids related to genes involved in oxidative phosphorylation and mitochondrial function. This was borne out in the functional Seahorse assays as well that showed significantly lower basal oxygen consumption rate, ATP production and non-mitochondrial oxygen consumption in schizophrenia organoids but not in bipolar disorder organoids. There have been a number of studies examining mitochondrial function in schizophrenia and bipolar disorder, many involving post-mortem brain studies, peripheral cells and neuroimaging [39–43]. Previous studies in bipolar disorder have shown decreased expression of electron transport chain components in the cortex [44, 45] as well as decreased mitochondrial respiration and increased glycolytic capacity [46, 47]. Our gene expression results as well as the Seahorse experiments point to disease-specific deficits of mitochondrial function in cerebral organoids from schizophrenia, possibly pointing to unique differences in mitochondrial function in the context of neurodevelopment in schizophrenia. Our findings of differences in mitochondrial respiration in schizophrenia cerebral organoids is consistent with a large body of literature suggesting deficits in the oxidative phosphorylation system in schizophrenia [48].

The pathways that were enriched in the differentially regulated genes in bipolar disorder organoids vis-à-vis schizophrenia organoids involved categories involved in ion binding and regulation of transport. Further examination of mitochondria–ER interactions showed that cortical neurons from bipolar disorder subjects showed a significant reduction MAMs compared to control cortical neurons but the MAMs in schizophrenia cortical neurons were in the same range as the control cortical neurons. ER stress has been hypothesized to play in important role in bipolar disorder [49, 50]. Studies have also indicated that the mood stabilizers lithium and valproic acid may mediate their therapeutic effects through modulation of pathways involved in ER stress [51, 52]. While various aspects of mitochondrial biology have been hypothesized to play a role in a schizophrenia and bipolar disorder [53, 54], our findings of disease-specific differences in ER–mitochondria interactions in bipolar disorder vis-à-vis schizophrenia is of interest in light of these previous studies. Altered ER–mitochondrial interactions may be an underlying mechanism that leads to the abnormal form and distribution of the mitochondrial network observed in cells from patients with bipolar disorder [39].

Our findings are also of interest in the context of a recent study analyzing transcriptional data for mitochondria-related gene expression the dorsolateral prefrontal cortex in post-mortem brain specimens from subjects with schizophrenia and bipolar disorder [55]. This study showed that schizophrenia and bipolar disorder had distinct differences in the nature of mitochondrial dysfunction, with a greater subset of genes that were differentially expressed in schizophrenia compared to bipolar disorder. Moreover, they found that the schizophrenia-related DEGs were enriched for mitochondrial function pathways involved in energy production while there was no such enrichment for bipolar disorder-related DEGs [55]. Our findings of mitochondrial function differences in schizophrenia organoids but not in bipolar disorder organoids are consistent with those post-mortem findings. Moreover, our experiments with the cortical neurons suggest that the mitochondria-related deficits in bipolar disorder may primarily result from aberrant mitochondria–endoplasmic reticulum interactions rather than mitochondrial pathways involved in energy production.

Among other pathways implicated by the DEGs in our transcriptomic analyses were those related to ion channel biology and neuro-transporters. Ion channel genes have been implicated in the disease biology of bipolar disorder and some therapeutic approaches have been tried based on modulation of ion channels [56, 57]. Genetic studies of rare variants in bipolar disorder have also suggested that these risk variants are enriched for genes that are related to the regulation of neuronal excitability [58]. Studies have suggested a role of excitatory/inhibitory (E/I) synaptic imbalance in bipolar disorder as well as in schizophrenia [59, 60]. In our functional studies neuronal firing using multi-electrode arrays that we have previously published, we found no differences in spontaneous neuronal firing in bipolar disorder or schizophrenia organoids when compared to healthy control organoids [20, 21]. However, these experiments had also shown that there was a significant reduction in the response to electrical stimulation and to KCl-induced depolarization in both bipolar disorder and schizophrenia organoids, suggesting some shared functional deficits in relation to neuronal excitability [20, 21].

While our studies add to the body of literature examining biological features that are shared and distinct between the disorders, there are a number of caveats to the results described in our study. The generation and culturing of organoids for 6–9 month periods introduces the possibility of stochastic variations that may affect the differentiation process. We have attempted to minimize such confounding factors by undertaking the organoid generation and culturing of the different groups in parallel and managed by the same researcher to avoid user-dependent and batch effects. Since schizophrenia and bipolar disorder are idiopathic disorders with complex genetics, we would ideally like to have a much larger sample size. However, given the time, labor and resource intensiveness of the cerebral organoid generation process makes it difficult at this time. Generating and studying a total of 24 cerebral organoids over the course of 9 months was a significant undertaking. While cerebral organoids capture some elements of the human brain biology, there are many limitations in our attempts to recapitulate the biology in the brain. While cerebral organoids contain many neuronal and glial cell types, including mature neurons and synaptic connections, they lack a vasculature in the way cerebral organoids are currently generated. In addition, the transcriptomic patterns of cerebral organoids most closely resemble the gene expression patterns observed in the fetal cortex. Hence, there are limitations in the study of psychiatric disorders that usually have their onset in young adulthood using cellular models that resemble the biology of the fetal brain. Hence, these models may have more relevance in understanding some of the neurodevelopmental factors or vulnerabilities that may be present long before the clinical manifestation of the illnesses. Along those lines, these cellular models may also enable the testing of specific perturbations that may intersect with the underlying complex genetic risk to unmask aberrant pathway as related to the disease biology during neurodevelopment [31, 32].

## Supplementary Information

Below is the link to the electronic supplementary material.

Supplementary file1 (XLSX 34629 KB) Cuffdiff analysis for the bipolar disorder vs schizophrenia.

Supplementary file2 (DOCX 61 KB) Tables.

## Data Availability

The data are available in NCBI's Gene Expression Omnibus had previously been deposited [20, 21] and are accessible through GEO Series accession number GSE134497 (https://www.ncbi.nlm.nih.gov/geo/query/acc.cgi?acc=GSE134497) and GSE133534 (https://www.ncbi.nlm.nih.gov/geo/query/acc.cgi?&acc=GSE133534).
