# Supplementary material for: Disease-specific differences in gene expression, mitochondrial function and mitochondria-endoplasmic reticulum interactions in iPSC-derived cerebral organoids and cortical neurons in schizophrenia and bipolar disorder
Source: Discov Ment Health. 2023 Mar 9;3(1):8. doi: 10.1007/s44192-023-00031-8 (PMC9998323; doi:10.1007/s44192-023-00031-8)
Supplement: Supplementary file 2 — Supplementary file2 (DOCX 61 KB) Tables. [file 44192_2023_31_MOESM2_ESM.docx]

Supplementary table 1: Information on subjects and iPSC lines used, as reported in previous publications^16-18^.
